# Evofosfamide for the treatment of human papillomavirus-negative head and neck squamous cell carcinoma 

**DOI:** 10.1172/jci.insight.169136

**Published:** 2023-02-22

**Authors:** Stephen M.F. Jamieson, Peter Tsai, Maria K. Kondratyev, Pratha Budhani, Arthur Liu, Neil N. Senzer, E. Gabriela Chiorean, Shadia I. Jalal, John J. Nemunaitis, Dennis Kee, Avik Shome, Way W. Wong, Dan Li, Nooriyah Poonawala-Lohani, Purvi M. Kakadia, Nicholas S. Knowlton, Courtney R.H. Lynch, Cho R. Hong, Tet Woo Lee, Reidar A. Grénman, Laura Caporiccio, Trevor D. McKee, Mark Zaidi, Sehrish Butt, Andrew M.J. Macann, Nicholas P. McIvor, John M. Chaplin, Kevin O. Hicks, Stefan K. Bohlander, Bradly G. Wouters, Charles P. Hart, Cristin G. Print, William R. Wilson, Michael A. Curran, Francis W. Hunter

Original citation: *JCI Insight*. 2018;3(16):e122204. https://doi.org/10.1172/jci.insight.122204

Citation for this corrigendum: *JCI Insight*. 2023;8(4):e169136. https://doi.org/10.1172/jci.insight.169136

It has been brought to the authors’ attention that the STR profile of the head and neck squamous cell carcinoma line UT-SCC-54C presented in Supplemental Table 1 is markedly different from that of UT-SCC-54A and UT-SCC-54B, which originated from the same patient. Upon further investigation, the authors have determined that the UT-SCC-54C cultures used in this study almost exclusively comprise colon cancer–derived HCT116 cells, as demonstrated by STR, gene expression, and mutation profiling. The authors regret contamination of the UT-SCC-54C line reported here and confirm that uncontaminated UT-SCC-54C cultures are available from earlier frozen stocks. The authors have stated that the conclusions of the study are not affected.

## Version 1 02/22/2023

Electronic publication

